# Has agricultural intensification impacted maize root traits and rhizosphere interactions related to organic N acquisition?

**DOI:** 10.1093/aobpla/plaa026

**Published:** 2020-06-19

**Authors:** Jennifer E Schmidt, Amisha Poret-Peterson, Carolyn J Lowry, Amélie C M Gaudin

**Affiliations:** 1 Department of Plant Sciences, University of California at Davis, One Shields Avenue, Davis, CA, USA; 2 USDA-ARS Crops Pathology and Genetics Research Unit, University of California, Davis, CA, USA; 3 Department of Natural Resources and the Environment, University of New Hampshire, 46 College Road, Durham, NH, USA

**Keywords:** Organic nitrogen, plant–microbe interactions, plasticity, rhizosphere, roots

## Abstract

Plant–microbe interactions in the rhizosphere influence rates of organic matter mineralization and nutrient cycling that are critical to sustainable agricultural productivity. Agricultural intensification, particularly the introduction of synthetic fertilizer in the USA, altered the abundance and dominant forms of nitrogen (N), a critical plant nutrient, potentially imposing selection pressure on plant traits and plant–microbe interactions regulating N cycling and acquisition. We hypothesized that maize adaptation to synthetic N fertilization altered root functional traits and rhizosphere microbial nutrient cycling, reducing maize ability to acquire N from organic sources. Six maize genotypes released pre-fertilizer (1936, 1939, 1942) or post-fertilizer (1984, 1994, 2015) were grown in rhizoboxes containing patches of ^15^N-labelled clover/vetch residue. Multivariate approaches did not identify architectural traits that strongly and consistently predicted rhizosphere processes, though metrics of root morphological plasticity were linked to carbon- and N-cycling enzyme activities. Root traits, potential activities of extracellular enzymes (BG, LAP, NAG, urease), abundances of N-cycling genes (*amoA*, *narG*, *nirK*, *nirS*, *nosZ*) and uptake of organic N did not differ between eras of release despite substantial variation among genotypes and replicates. Thus, agricultural intensification does not appear to have impaired N cycling and acquisition from organic sources by modern maize and its rhizobiome. Improved mechanistic understanding of rhizosphere processes and their response to selective pressures will contribute greatly to rhizosphere engineering for sustainable agriculture.

## Teaser

Synthetic nitrogen (N) fertilizers have fundamentally changed the availability of this critically important plant nutrient in agricultural systems, replacing organic sources such as compost and cover crops. Decades of plant breeding have created maize varieties that are highly productive under synthetic nitrogen fertilization, but potential trade-offs for uptake of organic nitrogen were unclear. Using a small panel of maize genotypes, we find minimal impacts of modern breeding on maize root traits, interactions between roots and associated microorganisms that regulate organic matter breakdown and transformations, and uptake of organic nitrogen from cover crops.

## Introduction

Agricultural intensification, particularly the increasing use of synthetic nitrogen (N) fertilizers, created fundamental shifts in how maize (*Zea mays*) is cultivated in the USA. Application of inorganic N has increased >40-fold since the introduction of these fertilizers, from 2 kg ha^−1^ in 1940 to 90 kg ha^−1^ in 2015 (Cao *et al.* 2018), replacing more sustainable forms such as organic N inputs from manure and cover crops. Breeders responded to the novel maize cultivation environment by developing new varieties that are highly productive in such intensively managed systems, due in part to changes in root traits and inorganic N acquisition (Duvick 2005; York *et al.* 2015). Nonetheless, it remains unclear whether adaptation to systems fertilized with inorganic N has caused a trade-off negatively impacting root traits and rhizosphere interactions facilitating acquisition of organic N.

Interactions between plant roots and soil microorganisms shape the dynamics of mineralization and nutrient-cycling processes that are the foundation of sustainable agricultural productivity. When organic matter is introduced to the soil, microbial communities produce extracellular enzymes to break down complex compounds further mineralized into plant-available inorganic ions which can be assimilated by roots or microorganisms or transformed to other forms of N through nitrification and denitrification. Plant root effects on these microbially mediated processes, or rhizosphere effects, range from facilitation and synergy to competition for substrates. Rhizodeposition, including both exudation of organic compounds and sloughing of root cells, provides carbon (C), N and other nutrients to fuel microbial metabolism. Targeted root proliferation in long-lasting resource patches such as slowly mineralizing cover crop residue thus enhances microbial transformations already occurring there and leads to increased plant resource acquisition (Mou *et al.* 2013).

Host plant influence on rhizosphere microbiome (rhizobiome) composition and functions regulating C and N cycling may be linked to specific root architectural, morphological and physiological traits, or microbiome-associated phenotypes (Oyserman *et al.* 2018). For instance, plant N uptake per unit root length in various plant species is related to rhizosphere bacterial community composition, with higher N uptake correlated with lower abundance of nitrate-reducing bacteria (Bell *et al.* 2015; Moreau *et al.* 2015). In grasslands, root diameter, dry matter content and C:N ratio can predict microbial N-cycling functions (Legay *et al.* 2014), while root morphology and chemistry are related to microbial biomass, C/N ratio and production of C-cycling enzymes that predict rates of C cycling (Carrillo *et al.* 2017). However, little is known about the influence of root functional traits on C- and N-cycling functions in major crop plants and downstream impacts on N acquisition in agroecosystems.

Resource acquisition and use efficiency in major crops are likely jointly regulated by plants and the rhizobiome. Plant nitrogen use efficiency (NUE), defined as the product of the plant physiological parameters N uptake efficiency and N utilization efficiency (Moll *et al.* 1982), has been linked to variation in rhizobiome composition and functions. High-NUE maize and rice lines have higher rhizobacterial diversity and abundance of proteolytic genes (Baraniya *et al.* 2016), distinct rhizosphere meta-transcriptomes (Pathan *et al.* 2018), stronger effects on rates of key nutrient-cycling enzymes (Pathan *et al.* 2015) and higher rhizosphere nitrification activity and abundance of ammonia-oxidizing bacteria (Li *et al.* 2008) compared to low-NUE lines. Identifying crop genotypes that facilitate rapid transformation of organic N to plant-available forms in the rhizosphere thus holds potential to increase nutrient use efficiency (Zhang *et al.* 2010; Shen *et al.* 2013) and decrease N losses from agroecosystems.

However, plant–rhizobiome interactions are highly context-dependent (Schmidt *et al.* 2019), so it is essential not only to identify genotypes that promote efficient microbial nutrient transformations, but also to understand how their roots and rhizosphere effects respond to the selective pressures imposed by agricultural management. The intensification of maize agricultural systems in the USA, particularly the increasing use of synthetic N fertilizers, represents a selective pressure that has affected both maize and its associated rhizobiome (York *et al.* 2015; Emmett *et al.* 2018; Schmidt *et al.* 2020). As modern breeding sought to create new hybrids adapted to systems with high rates of synthetic N fertilization, root architecture and anatomy shifted towards a more efficient N-acquiring phenotype (York *et al.* 2015) and further genetic and physiological improvements increased inorganic N uptake and uptake efficiency (Haegele *et al.* 2013; Emmett *et al.* 2018). Root morphology and morphological plasticity (shifts in proliferation and morphology in response to resource availability) may also have been affected, as intensively managed systems with high N availability favour unique root traits and banded fertilizer application may reduce the need for plasticity (Mi *et al.* 2010, 2016; Schmidt and Gaudin 2017).

**Figure 1.**
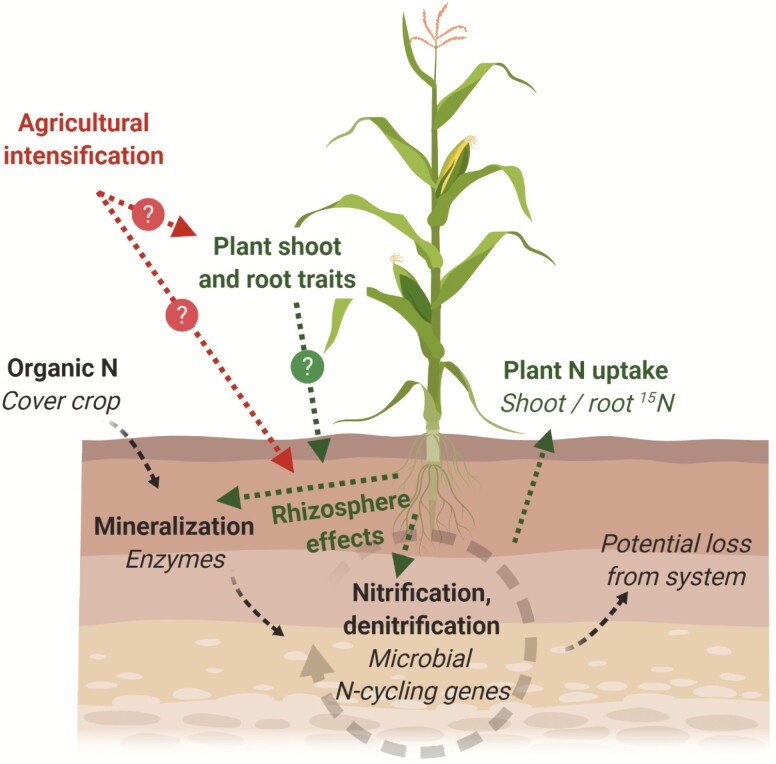
Potential effects of agricultural intensification on maize root traits and rhizosphere processes. Organic N (e.g. cover crops) added to agricultural soils undergoes microbially mediated processes of mineralization, ammonification, nitrification and denitrification. Plant rhizosphere effects influence the rates and dynamics of these processes through diverse mechanisms such as providing root exudates for microbial consumption and competing with microorganisms for inorganic N. We hypothesized that (i) some maize shoot and root traits are linked to the ability of individual genotypes to stimulate microbial C/N cycling; (ii) agricultural intensification may have altered maize root architectural and morphological traits related to organic N acquisition; and (iii) agricultural intensification may have negatively impacted maize ability to stimulate rhizosphere microbial C/N cycling. Hypotheses are indicated with question marks.

We asked whether the nearly 20-fold increase in synthetic N fertilizer use since 1940 has produced maize genotypes with high productivity under those conditions at the expense of root morphological traits involved in resource mining, the ability to promote rhizosphere interactions related to N cycling and N acquisition from organic sources. Six well-characterized genotypes released pre- or post-1942 were grown in rhizoboxes to measure root morphological plasticity, extracellular enzyme activity, microbial genes related to inorganic N cycling and N uptake from a patch of ^15^N-labelled cover crop residue. We hypothesized that (i) maize root architectural and morphological traits have changed as a consequence of adaptation to inorganic N fertilizers; (ii) maize ability to stimulate rhizosphere microbial C/N cycling and acquire N from organic sources has been negatively impacted by agricultural intensification; and (iii) genetic variation in maize rhizosphere effects (the ability to stimulate microbial C/N cycling) can be traced to a subset of root and shoot traits (Fig. 1). Better understanding of how adaptation to inorganic N may have impacted rhizosphere transformations and plant uptake of organic N can contribute to the development of resource-efficient maize cultivars and sustainable nutrient management strategies.

## Methods

### Rhizobox preparation

Soil was collected from the upper 15 cm of a certified organic maize field in Davis, CA after harvest in September 2017. An organically managed field with potential legacy effects of past cover crop plantings was chosen because the experiment was designed to test stimulation of microorganisms involved in breakdown of cover crop residue. Field soil was dried at 60 °C for 72 h and ground to pass a 2-mm sieve, then stored at 25 °C. Soil properties and the corresponding method citations can be found in **Supporting Information—Table S1**.

Soil was mixed 1:1 (v/v) with sand (The QUIKCRETE Companies, Columbus, OH, USA). A detailed description of rhizobox preparation with written and video protocols can be found in Schmidt *et al.* (2018). Briefly, rhizoboxes were constructed out of two panes of clear acrylic (61 cm tall × 40.5 cm wide) separated by 0.25 cm thick spacers **[see****Supporting Information—Fig. S1****]**. Each box was filled to 2.5 cm from the top with 1750 g of the soil:sand mixture, which was equivalent to 976 g sand and 774 g soil. An organic N treatment patch (35 g of the soil:sand matrix mixed with 1 g dried ^15^N-labelled legume residue mixture of clover and vetch, 1.33 at.% ^15^N) and control patch (35 g soil:sand matrix) were located 5 cm from the sides of the box and 25 cm deep. Soil was unfertilized outside of the organic N treatment patch.

### Greenhouse experiment and experimental design

Six maize hybrids were used in this study. Three double-cross hybrids released pre-1942, before inorganic N fertilizer use became widespread, were compared to three single-cross hybrids released post-1980, when inorganic N fertilizer use had peaked **[see****Supporting Information—Table S2****]**. Seeds were surface-sterilized by stirring in a 5 % NaOCl solution for 1 min and rinsing thoroughly with deionized (DI) water, then germinated 72 h on moist laboratory towelettes. Boxes were watered homogeneously with 100 mL DI water and one germinated seed per box was transplanted 2.5 cm deep in the centre of each box. The locations of seed, control patch and treatment patch were traced on the bottom acrylic pane. Boxes were supported at a 45° angle from horizontal so that gravitropism would cause roots to grow against the bottom pane. Light deprivation sheeting with a white exterior and black interior surface (Americover, Escondido, CA, USA) was wrapped around each box. Rhizoboxes were maintained in a greenhouse with natural lighting and watered by hand to 60 % water-holding capacity every 3 days. The experiment was arranged as a complete randomized block design with four replicates.

### Shoot and root functional traits

Plants were harvested at 25 days after transplanting. Shoots were clipped at the first crown root (closest to the top of the soil), dried at 60 °C to constant mass, weighed to record shoot dry weight (SDW) and ground with a mortar and pestle to pass a 2-mm sieve. Roots and rhizosphere soil within treatment and control patches were separated from the surrounding soil by cutting around the patches with a razor and removing the patch interior. Roots were recovered by passing patch soil through a 2-mm sieve, and subsamples of treatment and control patch soil were stored at 4 °C for extracellular enzyme analysis and −80 °C for quantitative PCR (qPCR) analysis of microbial N-cycling gene abundance. The remaining soil from the rhizobox was divided into rhizosphere soil (defined as adhering to roots) and bulk soil, and rhizosphere soil was passed through a 2-mm sieve so that remaining fine roots could be removed with tweezers. Gravimetric water content (GWC) was measured on ~30 g of both bulk and rhizosphere soil within 24 h of sampling. Soil was weighed and dried at 60 °C to constant weight; GWC was reported as g water per g dry soil (W.K. Kellogg Biological Station 2017).

After washing to remove soil particles, roots outside and inside the two patches were scanned separately using WinRhizo software (Pro version, Regent Instruments, Inc.) at a resolution of 1200 dpi on an Epson 11000XL scanner. WinRhizo was used to quantify total root length (TRL) and the length of roots belonging to the diameter classes <0.2, 0.2–0.4, 0.4–0.8, 0.8–1.6 and >1.6 mm (Chassot *et al.* 2001). All roots were then dried at 60 °C and weighed to record root dry weight (RDW). Root:shoot (R:S) ratio was calculated for each plant as


$$\begin{array}{l} R:S=Mass_{Dry\;\;roots}-Mass_{Dry\;\;shoot} \end{array}$$

Specific root length (SRL) was calculated for each plant as


$$\begin{array}{l} SRL=\frac{Totalrootlength}{Mass_{Dryroots}} \end{array}$$

The proportion of fine roots was calculated as


$$\begin{array}{l} \%f\;ineroots=\frac{Rootlengthin0-0.2mmsizeclass}{Totalrootlength} \end{array}$$

Root length densities (RLDs, cm root per cm^3^ soil) were calculated for the organic N treatment patch and control patch. Root responsiveness and foraging precision were calculated based on RLD as follows (Farley and Fitter 1999):


$$\begin{array}{l} Responsiveness=RLD_{OrgNPatch}-RLD_{ControlPatch} \end{array}$$

$$\begin{array}{l} Precision=\frac{RLD_{OrgNPatch}-RLD_{ControlPatch}}{RLD_{ControlPatch}} \end{array}$$

### Quantification of extracellular enzyme potential activity

Potential activity of the C- and N-cycling enzymes β-glucosidase (BG), *N*-acetyl-glucosaminidase (NAG) and leucine aminopeptidase (LAP) was quantified using fluorescence (Bell *et al.* 2013). Potential activity of urease was quantified using a protocol modified from Kandeler and Gerber (1988), with the colorimetric determination of ammonium according to Doane and Horwath (2003). Enzyme activities were reported per g dry soil by correcting for GWC of rhizosphere soil.

Percent upregulation of enzyme potential activities in the organic N treatment patch was calculated as


$$\begin{array}{l} \frac{Activity_{OrgNPatch}-Activity_{ControlPatch}}{Activity_{ControlPatch}}\times 100\% \end{array}$$

### qPCR of microbial gene abundances

Genes related to bacterial abundance (16S rRNA), ammonification (*amoA*) and nitrification (*narG*, *nirK*, *nirS* and *nosZ*) were quantified using qPCR with technical triplicates. Samples were amplified on an Applied Biosystems QuantStudio 6 Flex Real-Time PCR System in a reaction volume of 10 µL (1 µL template DNA), 5 µL Brilliant III Ultra-Fast SYBR Green QPCR Master Mix (Agilent Technologies) with 0.08 µL bovine serum albumin, 1 µL each of the forward and reverse primers (10 µmol L^−1^), 0.15 µL reference dye (Agilent Technologies) and 1.77 µL nuclease-free water. Quantitative PCR conditions were optimized for each bacterial gene and run with an initial denaturation at 95 °C for 3 min, followed by 35–50 cycles of denaturation at 95 °C for 30 s, annealing at a predetermined primer-specific temperature for 30 s, extension at 72 °C for 30–60 s and a final extension at 72 °C for 5 min (Table 1).

**Table 1.** Primers and reaction conditions for quantitative PCR.

Gene	Primers	Reaction conditions
16S	515F/801R (Caporaso *et al.* 2011)	56 °C annealing, extension 60 s
amoA	amoA_1F_bact/amoA_2R_bact (Rotthauwe *et al.* 1997)	60 °C annealing, extension 60 s, 35 cycles
narG	narG_f_Bru/narG_r_Bru (Bru *et al.* 2007)	58 °C annealing, extension 30 s, 40 cycles
nirK	F1aCu_nirK/R3Cu_nirK (Throbäck *et al.* 2004)	57 °C annealing, extension 60 s, 50 cycles
nirS	cd3aF_nirSR3cd_nirS (Throbäck *et al.* 2004)	57 °C annealing, extension 60 s, 35 cycles
nosZ	nosZ_F/nosZ_162ZR (Throbäck *et al.* 2004)	53 °C annealing, extension 60 s, 35 cycles

Copy number was calculated from a standard curve using QuantStudio™ Real-Time PCR Software. To correct for variation in efficiency between runs, data were divided by run efficiency as calculated from the slope of the standard curve. Gene abundances were reported per g dry soil by correcting for GWC of rhizosphere soil. Percent upregulation of gene abundance in the organic N treatment patch was calculated as for enzyme activities.

### ^15^N uptake

Organic N treatment patch roots were ground to pass a 2-mm sieve for ^15^N analysis and root and shoot samples were submitted for elemental analysis-isotope ratio mass spectrometry (EA-IRMS) at the UC Davis Stable Isotope Facility (Davis, CA, USA). N uptake from the label was calculated for shoots and roots from ^15^N at.% using the isotope dilution equations derived in Barraclough (1995).

### Multivariate statistical analyses

All statistical analyses were conducted in R software (v 3.6.3), and the data and code used for analysis are presented in **Supporting Information—Files S2****–****S7**. Multivariate statistical methods were used to identify a suite of plant traits predictive of variation in rhizosphere microbial C/N-cycling functions. First, correlation analysis was used to examine relationships among the full set of plant and microbial variables. Pearson correlation coefficients were calculated based on raw data (i.e. from each biological replicate rather than summarized by genotype or era) and a heatmap was generated using the corrplot package in R (Wei and Simko 2017).

Principal components analysis (PCA), an unconstrained ordination method, was used to visualize relationships among plant and microbial variables. The variables previously investigated with correlation analysis were scaled and centred such that each variable had a mean of 0 and variance of 1, and PCA was conducted with the stats package (R Core Team 2020). A biplot of the results was generated using the factoextra package to colour-code variables according to the strength of their contribution to the PCA (Kassambara and Mundt 2020).

### Univariate statistical analyses

Multivariate analyses revealed that a subset of plant traits (SDW, RDW, R:S ratio, TRL, responsiveness and precision) were correlated with shoot and/or root ^15^N content. Effects of era of release and genotype (nested within era) on these traits as well as shoot and root ^15^N content were tested with analysis of variance (ANOVA) using block as a random effect. To assess whether agricultural intensification has affected maize ability to stimulate rhizosphere microbial functions, ANOVA was used to test the effects of era of release and genotype (nested within era) on the upregulation of potential enzyme activities and N-cycling gene abundances, again using block as a random effect. Data for NAG and the *nirS* gene were transformed (logarithmic and Box–Cox transformations, respectively) prior to analysis to achieve normality of residuals. The Benjamini–Hochberg correction was used to control the family-wise error rate at α = 0.05.

## Results

### Plant growth, biomass partitioning and root morphological plasticity

**Figure 2.**
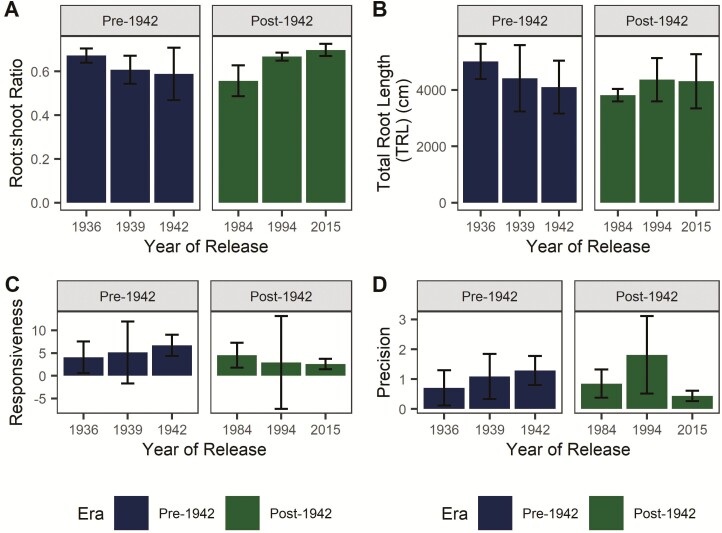
Plant root traits. (A) Root:shoot ratio and (B) TRL were calculated for each plant to assess potential differences in biomass partitioning and high-level root system traits. Root:shoot ratio was calculated as the ratio of dry root mass to dry shoot mass. (C) Responsiveness and (D) precision were calculated as metrics of root morphological plasticity. Responsiveness, calculated as the difference between RLD in the organic N patch relative to the control patch, tended to be slightly higher in older genotypes (*P* > 0.05). (B) Precision, defined as responsiveness normalized by RLD in the control patch, was not significantly different among genotypes (*P* > 0.05). Error bars represent standard error.

Plants had similar root and shoot biomass at harvest (era and genotype *P* > 0.05). Biomass partitioning was consistent between eras of release and among genotypes (*P* > 0.05), with R:S ratios of ~0.6 (Fig. 2A). Total root length was 1419–7073 cm and did not differ between eras of release or among genotypes (*P* > 0.05; Fig. 2B), and SRL did not differ between eras or among genotypes (all *P* > 0.05).

Morphological plasticity was observed in all root systems, with preferential root proliferation in the organic N treatment patch as indicated by intercepts significantly different from 0 in responsiveness and precision models. Responsiveness to organic N (RLD in the organic N treatment patch relative to the control patch) tended to be higher for genotypes released post-1940, but differences were not statistically significant at the α = 0.05 level (Fig. 2C). Precision, calculated as responsiveness normalized by RLD in the control patch, tended to vary among genotypes but era and genotype effects were not significant (all *P* > 0.05; Fig. 2D).

### Potential activity of extracellular enzymes

**Figure 3.**
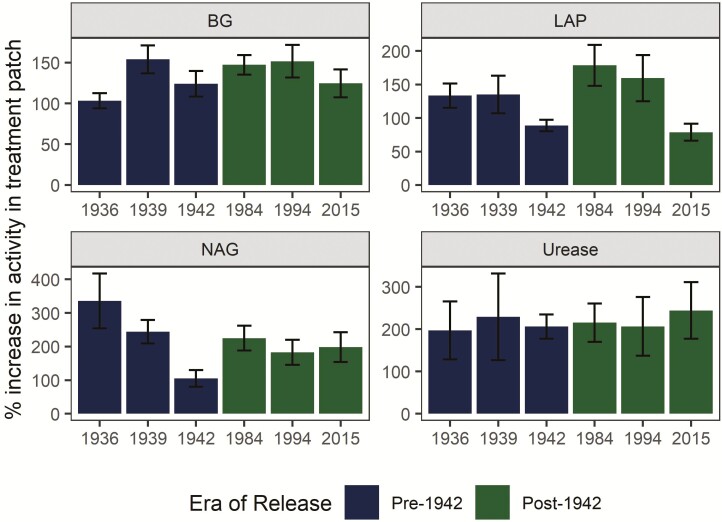
Upregulation of potential extracellular enzyme activity in the treatment patch. The potential activity of the C/N-cycling extracellular enzymes BG, LAP, NAG and urease was upregulated in the organic N treatment patch relative to the control patch. The degree of upregulation was not affected by genotype or era of release (Benajmini–Hochberg-adjusted *P* > 0.05). Error bars represent standard error.

The potential activity of all enzymes measured was upregulated in the treatment patch relative to the control (Fig. 3). However, the degree of upregulation was not significantly affected by era of release or genotype (Benjamini–Hochberg-adjusted *P* > 0.05). The potential activity of the C-cycling enzyme BG was ~100 % higher in the treatment patch, while the potential activity of urease was roughly 200 % higher in the presence of organic N.

### N-cycling genes

**Figure 4.**
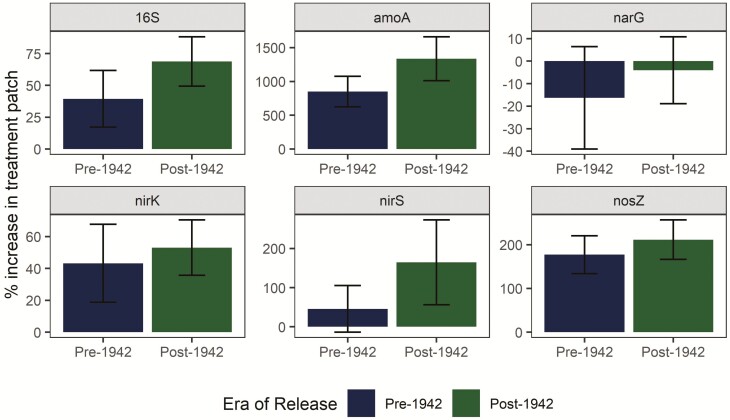
Upregulation of N-cycling gene abundances in the treatment patch. Genes related to nitrification (*amoA*) and denitrification (*nirK*, *nirS*, *nosZ*) were more abundant in the organic N treatment patch for all genotypes, but total bacterial abundance (16S rRNA) and *narG* gene abundance were not increased in the treatment patch. Upregulation in the patch tended to be higher in newer genotypes, although the trend was not statistically significant for any gene (all *P* > 0.05). Error bars represent standard error.

Abundance of four genes related to nitrification and denitrification was higher in the organic N patch than the control, but the abundance of the 16S rRNA gene and the *narG* gene were not significantly different between patches (Fig. 4). The *amoA* gene was most strongly upregulated in the organic N treatment patch, increasing in abundance by nearly 1000 % relative to the control patch. Upregulation in the treatment patch tended to be higher in genotypes released post-1940, although genotype and era effects were not statistically significant due to substantial variation between replicates (*P* > 0.05).

### N uptake from ^15^N-labelled cover crop residue

**Figure 5.**
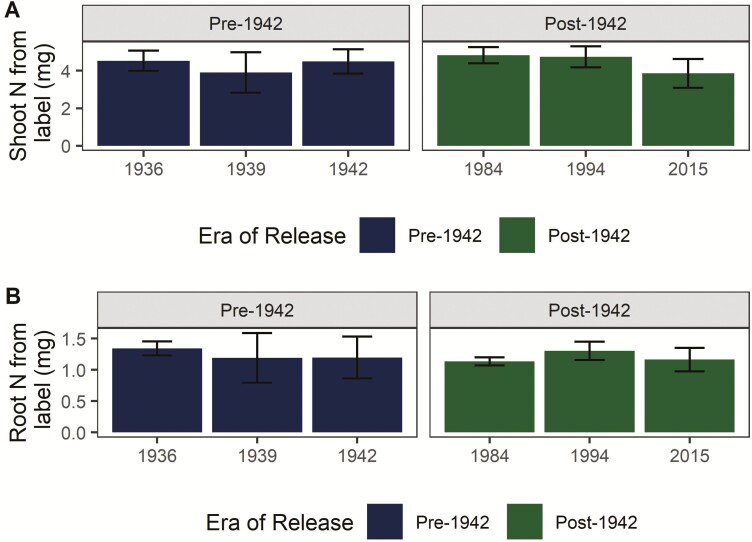
N uptake from ^15^N-labelled cover crop residue. The majority of N from the organic N source was localized in (A) shoot biomass at harvest, with (B) a smaller proportion partitioned to the root system. Uptake of N from cover crop residue did not vary among genotypes or by era of release (all *P* > 0.05). Error bars represent standard error.

Roughly 80 % of N taken up from the ^15^N-labelled clover and vetch residue was localized in shoots at harvest, with the remainder localized in the root system. N uptake from the label did not vary by era of release or genotype (*P* > 0.05; Fig. 5).

### Correlation analysis

**Figure 6.**
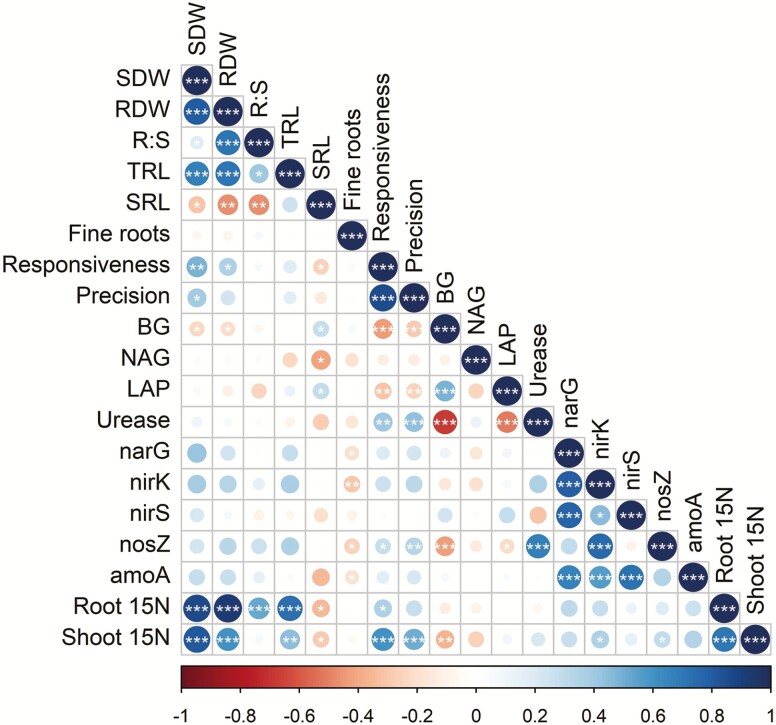
Correlations among plant and microbial variables. Correlations among plant traits and rhizosphere microbial C/N-cycling processes and plant N uptake from a ^15^N-labelled organic source. Plant traits included SDW; RDW; R:S ratio; TRL; SRL; fine roots, the percentage of roots >0.2 mm in diameter; responsiveness, the difference in RLD in the organic N treatment patch relative to the control patch; and precision, responsiveness normalized by RLD in the control patch. Microbial processes (BG; NAG; LAP; urease; *narG*; *nirK*; *nirS*; *nosZ*; and *amoA*) were represented by upregulation of potential enzyme activity or gene abundance in the treatment patch relative to the control patch: (Value_treatment_ − Value_control_)/Value_control_ * 100. The magnitude and sign of Pearson correlation coefficients are indicated by the colour scale at the bottom. Stars indicate significant correlations (***P < 0.001; **P < 0.01; *P < 0.05).

Pearson correlation coefficients revealed that plant traits tended to be correlated more strongly than microbial functions with uptake of organic N from the treatment patch (Fig. 6). Strong positive correlations of shoot and root dry weight, TRL and R:S ratio with root and shoot ^15^N revealed that plant and root system size were the strongest predictors of organic N uptake from the treatment patch. Root responsiveness and precision were strongly correlated with one another and with shoot ^15^N (all *P* < 0.001), weakly positively correlated with SDW (*P* < 0.05), negatively correlated with BG and LAP (*P* < 0.01) and positively correlated with urease (*P* < 0.01). Specific root length was slightly negatively correlated with shoot and root ^15^N (both *P* < 0.05), while percentage of fine roots was slightly negatively correlated with microbial functions including *narG*, *nirK*, *nosZ* and *amoA* (all *P* < 0.05). BG and LAP were positively correlated with one another and negatively correlated with urease (all *P* < 0.001). Strong positive correlations were observed among nearly all microbial N-cycling genes, but these were not correlated with plant traits or ^15^N uptake from the label (Fig. 6).

### Principal components analysis

**Figure 7.**
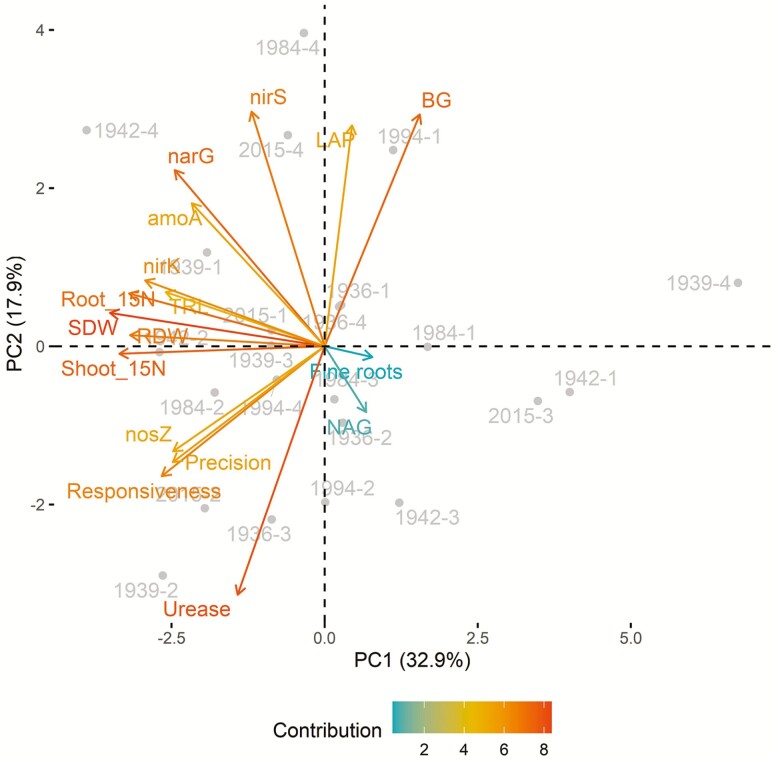
Principal components analysis biplot. Principal components analysis was conducted to examine relationships among plant and microbial variables and their effect on variation among experimental units. The first principal component (PC1) accounted for 33 % of variation among samples and the second principal component (PC2) accounted for only 18 % of variation. Arrows represent plant and microbial variables, with colour indicating the magnitude of a variable’s contribution to each principal component. Dots represent experimental units and are labelled with a combination of genotype year of release and biological replicate.

Principal components analysis was then used to visualize relationships among predictor variables and experimental units. The first and second principal components (PC1, PC2) accounted for 33 % and 18 % of variation among experimental units (Fig. 7). Variables with the strongest contribution to PC1 included SDW, RDW, root ^15^N and shoot ^15^N, while variables with the strongest contribution to PC2 included upregulation of urease, BG and *nirS*. Percentage of fine roots and upregulation of NAG activity had the lowest contributions to PC1 and PC2 of the variables investigated. Samples did not cluster by era of release or genotype.

## Discussion

We first asked whether agricultural intensification has affected maize root traits related to acquisition of N from organic sources (Fig. 1). No significant effects of era of release were observed on root traits including root biomass, R:S ratio, TRL, SRL and the proportion of fine roots (Fig. 2; Schmidt *et al.* 2018). Agricultural intensification has not decreased root morphological plasticity, as shown by strong proliferation of roots in the organic N patch despite a slight trend towards decreased responsiveness in newer genotypes (*P* > 0.05; Fig. 2C). Foraging precision, which influences nutrient uptake and the outcome of interspecific competitive interactions (Rajaniemi 2011), varied substantially among experimental units, with the highest precision and variability measured in a maize hybrid released in 1994 (Fig. 2D). Despite changes in the form and abundance of N due to synthetic fertilizers, root system architecture did not differ between eras of release in these hybrids, and the lack of between-era differences in root morphological plasticity suggests that exploitation of resource-rich patches in the heterogeneous soil environment has remained an important trait.

Consistent upregulation by all genotypes of potential enzyme activities and N-cycling gene abundances in the organic N patch (Figs 3 and 4) did not provide support for our third hypothesis, that agricultural intensification has negatively impacted maize stimulation of rhizosphere C/N-cycling processes (Fig. 1). Enzyme and gene abundances may have been driven primarily by the increased resource availability to microorganisms in the organic N patch and only minimally influenced by plant genotypes (i.e. differences in the ability of maize genotypes to stimulate rhizosphere microbial processes). Microorganisms can release extracellular enzymes in response to nutrient limitation (Allison and Vitousek 2005) and microbial N transformations has been shown to increase in a root-free localized N patch as compared to non-N-enriched soil (Wang *et al.* 2010). N availability was likely equivalent across rhizoboxes for the duration of the experiment, as N content of the treatment patch was standardized at start of the experiment, root growth rates did not vary among genotypes (Schmidt *et al.* 2018) and total uptake from the patch was equal at the end of the experiment (Fig. 5). It is possible that genotypic differences in N uptake would be magnified later in the season when N sink demand is greater due to tissue and grain development, especially given increased N uptake in newer hybrids noted elsewhere (Emmett *et al.* 2018).

Contrary to our third hypothesis (Fig. 1), multivariate analysis did not identify plant root and shoot traits that were strong and consistent predictors of microbial C/N cycling. Only metrics of root morphological plasticity (responsiveness and precision) were correlated with upregulation of extracellular enzyme activities and N-cycling gene abundances (Fig. 6) or aligned with those variables in the PCA biplot (Fig. 7). However, these correlations did not translate to improved resource capture from the organic N patch, a result also noted elsewhere (Hodge *et al.* 2008). Plant SDW and high-level root architectural traits were positively correlated with N acquisition from an organic source, but not with rhizosphere microbial functions (Fig. 6). Metrics related to root diameter distribution (SRL and percentage of fine roots), though linked elsewhere to rhizosphere N availability and rhizobiome composition (Corneo *et al.* 2016), were not important predictors of rhizosphere C/N cycling and organic N acquisition in this study. It may be that significant correlations to microbial processes would emerge only among genotypes with greater contrast in root architectural and morphological traits. Although modern breeding has affected root architectural and anatomical phenes in other maize genotypes (York *et al.* 2015), it does not appear to have altered the proportion of fine versus coarse roots in these genotypes (Schmidt *et al.* 2018). Root traits not measured here may additionally influence the rhizobiome, particularly exudate quantity and quality, known drivers of plant–microbe interactions (Berg and Smalla 2009).

Modern hybrids proliferate roots in response to organic N patches, stimulate microbial mineralization and transformation processes and acquire N from cover crop residues as efficiently as older hybrids at early developmental stages. These findings have profoundly positive implications for sustainable intensification of agroecosystems, showing that increasing soil organic matter could contribute to meeting crop nutrient demand in addition to numerous co-benefits such as C sequestration, reduced erosion and improved drought resistance and resilience (Lal *et al.* 2015; Paustian *et al.* 2016). Nonetheless, critical knowledge gaps impede our ability to translate this research into practice. Our results suggest distinct plant and microbial responses to agricultural intensification rather than coordinated adaptation. Understanding of plant–microbiome co-adaptation and co-evolution in agricultural systems remains limited, particularly the unique mechanisms and time frames that govern adaptation to selective pressure in plants and microorganisms. The mechanisms and timing of development of microbiome-associated phenotypes are another grey area; high-throughput methods such as host–microbiome genome-wide association studies (Awany *et al.* 2018) may aid in clarifying the genetic basis of some rhizosphere interactions and facilitating their integration into breeding programmes. Despite these research gaps, rhizosphere engineering strategies that maximize beneficial plant–rhizobiome interactions continue to hold promise for improving resource use efficiency in agricultural systems.

## Supporting Information

The following additional information is available in the online version of this article—

File S1. Figure S1: Layout of rhizoboxes. Rhizoboxes were filled with a 1:1 (v/v) soil:sand mixture and contained two patches. The organic N treatment patch contained 1 g ^15^N-labelled clover/vetch residue mixed with 35 g of the soil:sand mixture and the control patch contained 35 g of the soil:sand mixture. Germinated seeds were planted 2.5 cm from the top of the box with the radicle oriented downwards.

File S2. This file contains the code used for analyses in this manuscript.

File S3. This file contains plant and soil data.

File S4. This file contains plant, enzyme and gene abundance data in a format useful for multivariate analyses.

File S5. This file contains data for extracellular enzyme potential activities.

File S6. This file contains ^15^N data for plant shoot and root samples.

File S7. This file contains qPCR data for microbial gene abundances.

File S8. Table S1: Soil properties prior to mixing with sand.

File S9. Table S2: Genetic material used in this study.

plaa026_suppl_Supplementary_Figure_S1

plaa026_suppl_Supplementary_File_S2_S7

plaa026_suppl_Supplementary_Table_S1

plaa026_suppl_Supplementary_Table_S2

## Sources of Funding

This work was supported by the University of California, Davis and the College of Agricultural and Environmental Sciences through a Graduate Scholars Fellowship to J.E.S.; the Foundation for Food and Agriculture Research; and the USDA National Institute of Food and Agriculture, Agricultural Experiment Station Project (grant number CA-D-PLS-2332-H) to A.C.M.G.

## Conflict of Interest

None declared.
