# Supplementary figures and images for: Has agricultural intensification impacted maize root traits and rhizosphere interactions related to organic N acquisition?
Source: AoB Plants. 2020 Jun 19;12(4):plaa026. doi: 10.1093/aobpla/plaa026 (PMC7333546; doi:10.1093/aobpla/plaa026)

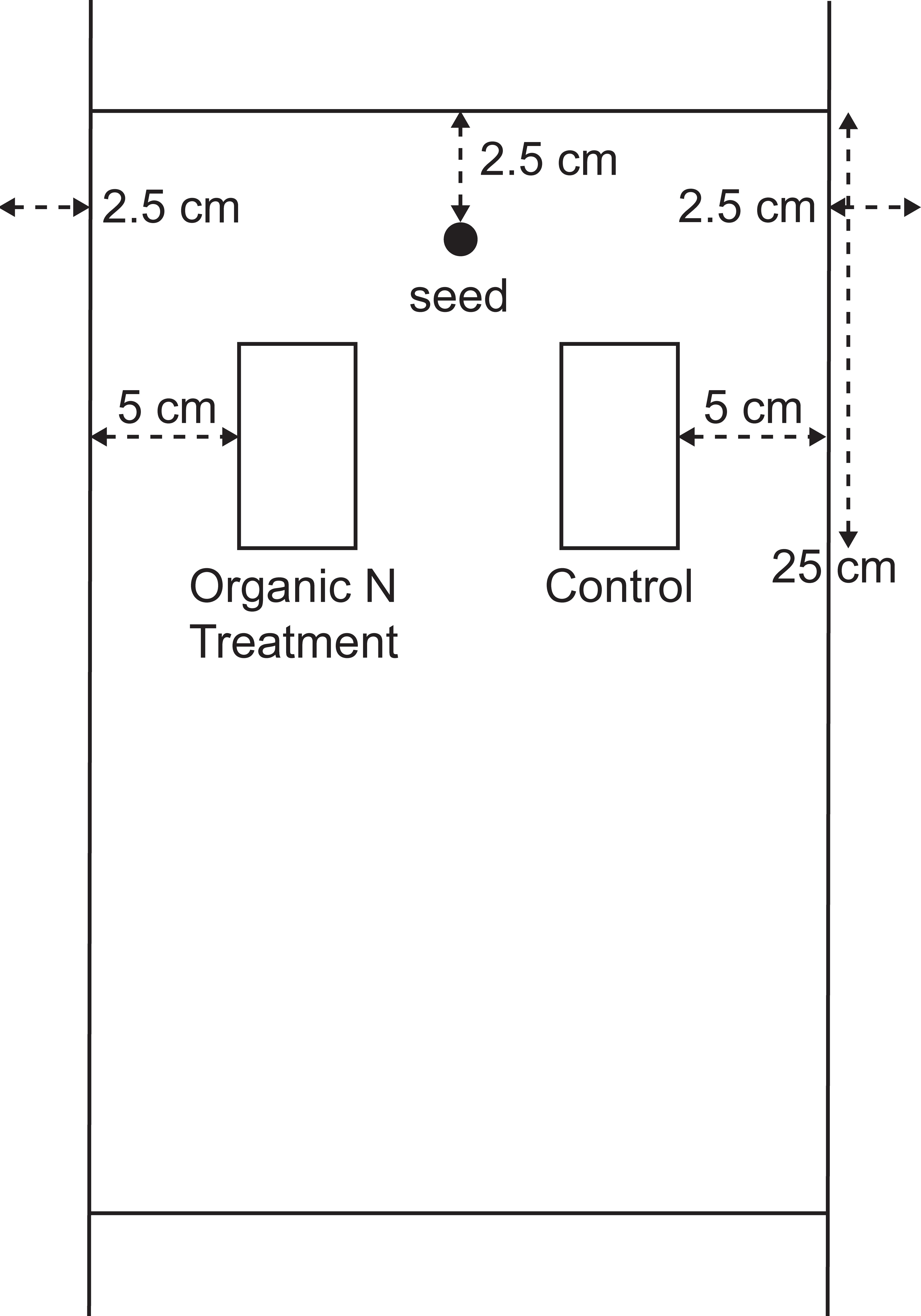

Supplement: plaa026_suppl_Supplementary_Figure_S1 [file plaa026_suppl_supplementary_figure_s1.jpeg]
