# Supplementary material for: Has agricultural intensification impacted maize root traits and rhizosphere interactions related to organic N acquisition?
Source: AoB Plants. 2020 Jun 19;12(4):plaa026. doi: 10.1093/aobpla/plaa026 (PMC7333546; doi:10.1093/aobpla/plaa026)
Supplement: plaa026_suppl_Supplementary_Table_S1 [file plaa026_suppl_supplementary_table_s1.docx]

|  | NO_3_-N | Olsen-P | X-K | X-K | X-Na | X-Na | X-Ca | X-Mg | CEC (estimated) | OM (LOI) | pH |
| --- | --- | --- | --- | --- | --- | --- | --- | --- | --- | --- | --- |
|  | *ppm* | *ppm* | *ppm* | *meq/100g* | *ppm* | *meq/100g* | *meq/100g* | *meq/100g* | *meq/100g* | *%* |  |
| Protocol reference | (Hofer 2003; Knepel 2003) | (Olsen and Sommers 1982; Prokopy 1995) | (Thomas 1982) | (Thomas 1982) | (Thomas 1982) | (Thomas 1982) | (Thomas 1982) | (Thomas 1982) | (Thomas 1982) | (Nelson and Sommers 1996) | (U.S. Salinity Laboratory Staff 1954) |
| Value | 177 | 50.6 | 557 | 1.42 | 224 | 0.98 | 8.60 | 10.6 | 21.6 | 3.10 | 7.53 |

Table S1: Soil properties prior to mixing with sand

References:

Hofer S (2003) Determination of Ammonia (Salicylate) in 2M KCl soil extracts by Flow Injection Analysis. Lachat Instruments, Loveland, CO

Knepel K (2003) Determination of Nitrate in 2M KCl soil extracts by Flow Injection Analysis. QuikChem Method 12-107-04-1-B. Lachat Instruments, Loveland, CO

Nelson DW, Sommers LE (1996) Total Carbon, Organic Carbon, and Organic Matter. In: Bigham JM (ed) Methods of Soil Analysis. Part 3. Chemical Methods. SSSA, Madison, WI, pp 1001–1006

Olsen SR, Sommers LE (1982) Phosphorus. In: Page AL (ed) Methods of soil analysis: Part 2. Chemical and microbiological properties. ASA and SSSA, Madison, WI, pp 1035–1049

Prokopy W (1995) Phosphorus in 0.5 M sodium bicarbonate soil extracts. Lachat Instruments, Milwaukee, WI

Thomas GW (1982) Exchangeable cations. In: Page AL (ed) Methods of soil analysis: Part 2. Chemical and microbiological properties. ASA, pp 159–165

U.S. Salinity Laboratory Staff (1954) pH reading of saturated soil paste. In: Richards LA (ed) Diagnosis and improvement of saline and alkali soils. U.S. Government Printing Office, Washington, D.C., p 102
