# Supplementary material for: Has agricultural intensification impacted maize root traits and rhizosphere interactions related to organic N acquisition?
Source: AoB Plants. 2020 Jun 19;12(4):plaa026. doi: 10.1093/aobpla/plaa026 (PMC7333546; doi:10.1093/aobpla/plaa026)
Supplement: plaa026_suppl_Supplementary_Table_S2 [file plaa026_suppl_supplementary_table_s2.docx]

Table S2. Genetic material used in this study

| Release date | Era of release | Type of hybrid | Supplier |
| --- | --- | --- | --- |
| 1936 | Pre-1942 | Double cross | Pioneer |
| 1939 | Pre-1942 | Double cross | Pioneer |
| 1942 | Pre-1942 | Double cross | Pioneer |
| 1984 | Post-1942 | Single cross | Pioneer |
| 1994 | Post-1942 | Single cross | Pioneer |
| 2015 | Post-1942 | Single cross | DeKalb |
